# Fabrication of Composite Beads Based on Calcium Alginate and Tetraethylenepentamine-Functionalized MIL-101 for Adsorption of Pb(II) from Aqueous Solutions

**DOI:** 10.3390/polym10070750

**Published:** 2018-07-06

**Authors:** Nan Wang, Li-Ye Yang, Yang-guang Wang, Xiao-kun Ouyang

**Affiliations:** School of Food and Pharmacy, Zhejiang Ocean University, Zhoushan 316022, China; ynwangnan@163.com (N.W.); liyey@zjou.edu.cn (L.-Y.Y.); wangyg0510@163.com (Y.-g.W.)

**Keywords:** sodium alginate, functional MIL-101, composite beads, adsorption, Pb(II)

## Abstract

In this work, a tetraethylenepentamine (TEPA)-grafted metal-organic framework material (MIL-101) was synthesized. The introduction of TEPA increased the abundance of functional groups on the MIL-101. As a powdery adsorbent, MIL-101-TEPA can be difficult to separate. In order to solve this problem, we combined MIL-101-TEPA with sodium alginate (SA) and injected the mixture into a CaCl_2_ solution to solidify the powder into beads with a particle size of 3 mm. The easily recovered adsorbent was applied to the adsorption of Pb(II) from water. The structure and characterization of the adsorbent were investigated through scanning electron microscope (SEM), Fourier-transform infrared spectroscopy (FTIR), X-Ray diffraction (XRD), thermogravimetric analysis (TGA), and X-ray photoelectron spectroscopy (XPS). We also optimized the adsorption conditions. The results of the study showed that the adsorption process was chemisorptive and endothermic in nature. The maximum adsorption capacity of the composite beads was 558.6 mg/g. Meanwhile MIL-101-TEPA@CA showed good repeatable utilization.

## 1. Introduction

Pb(II) is a kind of heavy metal that can enter the human body through gas, water, and food. Once the heavy metal Pb(II) enters the human body, it can be complexed with proteins and amino acids to affect metabolic function [1]. The long-term accumulation of Pb(II) in the body can cause Pb(II) poisoning, including symptoms of irritability or depression as well as nervous, cardiovascular, and pulmonary impairments [2,3]. In recent years, Pb(II) concentrations exceeding standard limits in both drinking water and liquid food have been reported [4]. Therefore, it is important to remove residual Pb(II) from drinking water and liquid food.

The adsorption method is the most commonly used to remove Pb(II) from liquids [5]. Adsorbents are usually dispersed in the liquid containing Pb(II) and are mixed with the liquid by stirring and concussion. After the adsorption is completed, the adsorbent is recovered by centrifugation or filtration. Traditional adsorbents include zeolites [6], activated carbon [7], resin [8], and other materials [9]. Among them, activated carbon is environmentally friendly, shows good adsorption properties, and adsorbs various metal ions and organic pollutants; it has been used as a commercialized adsorbent. Polymer adsorbents, including graphene oxide [10], carbon nanotubes [11], carbon nanotubes [12], and chitosan [13], also show good adsorption capacities for Pb (II). 

Metal-organic frameworks (MOFs) are a new type of porous materials [14], recently, MOFs have been widely used in the field of adsorption [15,16]. These materials are zeolites with networked structures, formed by the self-assembly of metal ions with organic compounds containing O or N elements [17]. MOF materials were first proposed by Yaghi et al [18]. MIL-101(Cr) is a kind of MOF with an octahedral structure and the chemical formula Cr_3_F(H_2_O)_2_O[BDC]_3_·*n*H_2_O. It has a high specific surface area, high water stability, and good thermal stability [19,20]. In addition, it can be modified simply. The Cr ion in MIL-101(Cr) provides unsaturated active metal sites, which can chelate electron-rich groups and permit the functionalization of MIL-101(Cr). Using this principle, the Férey group reported an ethylenediamine-modified MIL-101 for the first time [21]. Luo et al. applied ethylenediamine-modified MIL-101(Cr) to remove Pb(II) [22]. Amino groups also coordinate well with Pb(II); compared to MIL-101(Cr), the adsorption capacity of ethylenediamine-modified MIL-101 is greatly improved. 

MIL-101-TEPA shows a promising adsorption capacity for Pb(II). However, as a powdery adsorbent, MIL-101(Cr) is hard to capture in liquid. Sodium alginate (SA) is a natural polysaccharide, composed of β-d-mannuronic acid and α-l-guluronic acid [23]. When cations such as Ca^2+^ and Sr^2+^ are present in SA solution, the ion exchange reaction occurs between Na^+^ in α-l-guluronic acid and the bivalent cations [24]. A cross-linked network structure is formed by the accumulation of α-l-guluronic acid, which then forms a hydrogel. SA contains a large number of carboxyl groups and also shows pH sensitivity. Therefore, SA is widely used in the field of adsorption and medical treatment [25,26].

In this work, we have adopted tetraethylenepentamine (TEPA), a rich amine-containing chemical compound, to modify MIL-101(Cr). MIL-101-TEPA was used to adsorb Pb(II). SA was mixed with MIL-101-TEPA and the mixture was injected into a CaCl_2_ solution for solidification into MIL-101-TEPA@calcium alginate (CA) beads with a particle size of 3 mm. The larger-sized mixed adsorbent could be recovered by simple filtration.

## 2. Materials and Methods

### 2.1. Materials

Cr(NO_3_)_3_·9H_2_O, *p*-phthalic acid (BDC), CaCl_2_, hydrofluoric acid (HF), SA (200 ± 20 mPa·s), and TEPA were purchased from Aladdin Chemical Reagent Co., Ltd. (Shanghai, China). Pb(NO_3_)_2_, *N*,*N*-dimethylformamide (DMF), ethanol, and acetic acid were of analytical reagent grade and purchased from Sinopharm Chemical Reagent Co., Ltd. (Beijing, China). 

### 2.2. Preparation of MIL-101-TEPA@CA Beads

#### 2.2.1. Preparation of MIL-101

Férey’s group firstly synthesized MIL-101. In this work we adopted Férey’s scheme to prepare MIL-101. The reagent molar ratio of Cr(NO_3_)_3_·9H_2_O, BDC, HF, and H_2_O was 1:1:1:278. More concretely, 0.01 mol of Cr(NO_3_)_3_·9H_2_O and BDC were dispersed in 50 mL deionized water and processed ultrasonically to accelerate dissolution; 0.01 mol HF was then added to the above mixture. After 30 min continuous stirring, the mixed solution was transferred to a reaction kettle. The reaction temperature was 473 K and the reaction time was 8 h. The obtained product was washed three times alternately by DMF and ethanol. After washing, the product was placed in a vacuum drying box and dried for 24 h at 343 K.

#### 2.2.2. Preparation of MIL-101-TEPA

MIL-101-TEPA was prepared by the previously reported method [27]. Based on the experimental results, the method was adjusted and improved. After drying at 403 K, 0.6 g MIL-101 was dispersed into 60 mL anhydrous absolute ethanol, followed by the addition of 2 mmol TEPA. The final mixed solution was refluxed for 12 h at 363 K. The product was washed two times alternately in methanol and deionized water, after drying in a vacuum box at 373 K for 12 h, MIL-101-TEPA was obtained.

#### 2.2.3. Preparation of MIL-101-TEPA@CA Beads

SA solution with a concentration of 2% was named solution A. MIL-101 (0.2 g) was dispersed in a separate container with 10 mL deionized water as solution B. After ultrasonication for 20 min, equal volumes of solutions A and B were fully mixed. The mixed colloidal solution was extracted with a syringe and slowly dripped into 200 mL of a 2% CaCl_2_ solution for curing. In the process of dripping, the beaker of CaCl_2_ solution was stirred continuously using a magnetic agitator to accelerate the curing process and prevent the adhesion of the microspheres. After curing for 30 min, the remaining ions on the surfaces of the beads were washed by deionized water. 

Simple CA beads were also prepared by the following method: 0.4 g SA was dissolved in 20 mL deionized water, and the process of curing and washing was applied as in the preparation of the MIL-101-TEPA/CA beads. The prepared beads were placed in the drying box, and the temperature was held at 313 K for drying overnight. The dry weight of the MIL-101-TEPA/CA wet beads per gram was 32 mg; that of SA wet beads per gram was 32.26 mg.

### 2.3. Characterization

A Scanning electron microscopy (SEM, S4800, Hitachi Corp., Tokyo, Japan) and transmission electron microscopy (TEM, JEM-2100, Hitachi Corp., Tokyo, Japan) were used to record the surface morphology of the adsorbent. Energy-dispersive spectroscopy (GENESIS XM, EDAX Corp., New Castale, DE, USA) were used to analyze the element distribution. Fourier-transform infrared (FTIR) spectroscopy was conducted by a Tensor II spectrometer (Bruker Corp., Karlsruhe, Germany) to identify the surface groups of the adsorbent. X-ray photoelectron spectroscopy (XPS) was performed using AXIS ULTRA DLD, (Shimadzu, Tokyo, Japan). Thermogravimetric (TGA) analysis was performed using a TG/DTA (Perkin-Elmer, New Castale, DE, USA), and X-ray diffraction (XRD) was performed using a D8 Advance X-ray diffractometer (Bruker Corp., Karlsruhe, Germany).

### 2.4. Adsorption Experiment

The MIL-101-TEPA@CA beads were applied for the removal of Pb(II). The configuration of the Pb(II) mother liquid is as follows: 0.4 g Pb(NO_3_)_2_ was dissolved in 250 mL deionized water. This liquor was refrigerated at 277 K; each Pb(II) solution used in this experiment was diluted from the mother liquor. We compared the adsorption capacities of the adsorbents prepared in this work. In addition, the adsorption conditions were optimized, including the adsorbent dose, adsorption temperature, adsorption time, pH, and concentration of the Pb(II) solution. The balance relation between mass and time is shown in Equation (1), which was used to determine the adsorption amount of Pb(II). The balance relation between the mass and initial concentration is shown in Equation (2).

(1) $$q_{t}=\frac{(C_{0}-C_{t})V}{m}$$

(2) $$q_{e}=\frac{(C_{0}-C_{e})V}{m}$$

where *C*_0_ (mg/L) is the initial concentration, *m* (g) is the dose of MIL-101-TEPA@CA, *V* (L) is the volume of the Pb(II) solution, *C_t_* (mg/L) is the concentration of Pb(II) at a certain time, and *C_e_* (mg/L) is the Pb(II) concentration after adsorption has achieved equilibrium. The removal ratio (*R*) of Pb(II) is calculated by Equation (3):

(3) $$R=\frac{C_{0}-C_{e}}{C_{0}}\times 100\%$$

### 2.5. Reutilization Test

HCl with a concentration of 0.1 mol/L was applied to remove Pb(II) adsorbed by MIL-101-NH_2_@CA. In a typical removal process, 0.5 g of Pb(II)-loaded MIL-101-NH_2_@CA was placed in 50 mL eluent and shaken at room temperature for 4 h. This operation was repeated until no Pb(II) was detected in the desorbed eluate. The desorbed MIL-101-NH_2_@CA was reused five times and its adsorption capacity was measured after each use.

## 3. Results and Discussion

### 3.1. Synthesis of MIL-101-TEPA@CA

**Scheme 1:**
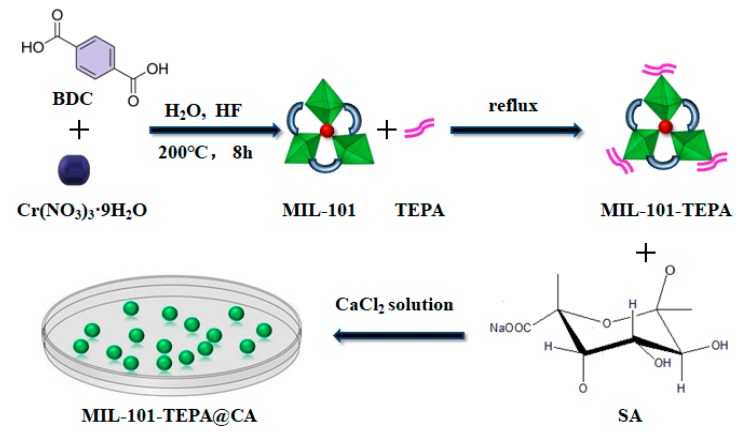
Preparation of MIL-101-TEPA@CA.

MIL-101 was synthesized by chromate and BDC in deionized water containing the mineralizer of HF. Abundant unsaturated active sites existed in the central metal Cr^3+^ of MIL-101. These sites could react with electron-rich groups such as ethylenediamine and TEPA, to realize functionalized MIL-101. Na^+^ in SA could be replaced by Ca^2+^; however, the resulting CA was insoluble in water. Based on this principle, after mixing the MIL-101-TEPA and SA solutions, MIL-101-TEPA@CA beads could be prepared in a CaCl_2_ solution. The process of preparing MIL-101-TEPA@CA is shown in Scheme 1. The best concentration of SA for bead formation is 1–2%; when the concentration of SA is greater than 1%, the viscosity of the SA/MIL-101-TEPA mixture impedes the formation of the regular beads. Therefore, the concentration of SA is set to 1%. The effect of the additive amount of MIL-101-TEPA on the adsorption efficiency of the composite beads was investigated, with results shown in Figure 4a. The adsorption performance of MIL-101-TEPA@CA for Pb(II) is increased with increases in the MIL-101-TEPA:CA mass ratio. When the mass ratio of MIL-101-TEPA:SA exceeds 1, the beads are irregular in globosity and easily broken. Thus, we set the mass ratio of MIL-101-TEPA and SA to 1:1.

### 3.2. Characterization

#### 3.2.1. SEM Observation

**Figure 1:**
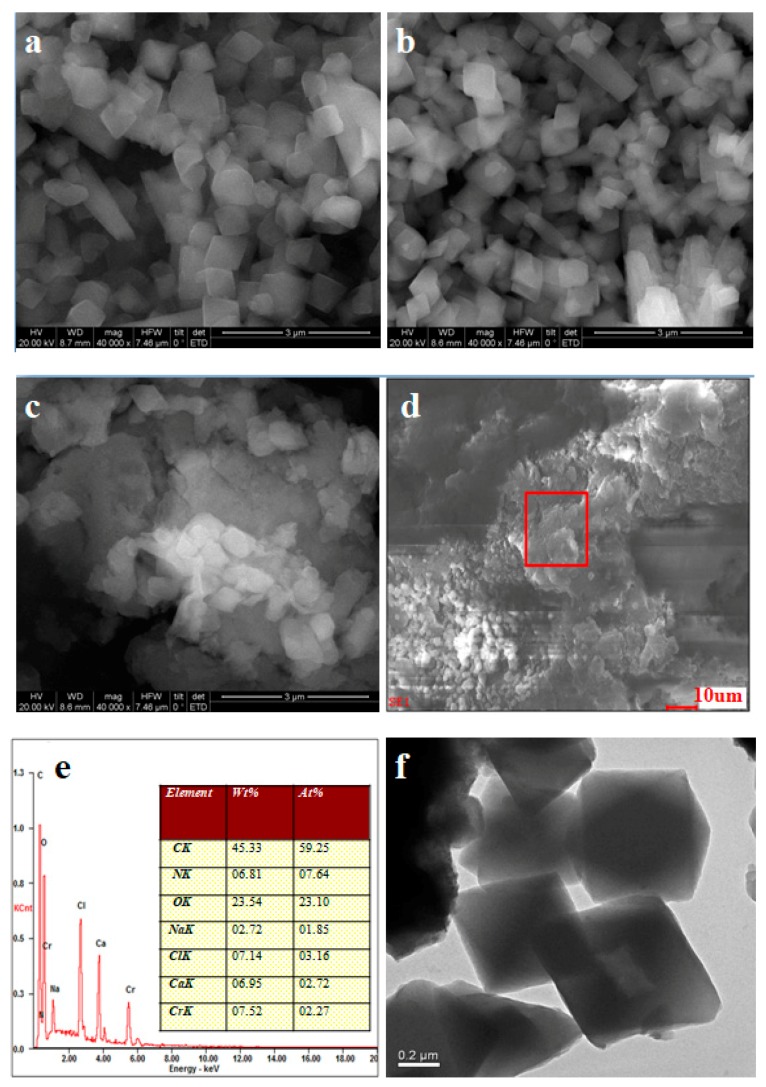
Scanning electron microscope (SEM) images of (**a**) MIL-101, (**b**) MIL-101-TEPA, and (**c**) MIL-101-TEPA@CA images at 3 μm; (**d**,**e**) EDS analysis; (**f**) TEM image of MIL-101-TEPA@CA at 2 μm.

The scale bar of all micrographs in Figure 1 is 3 μm. As shown in Figure 1a, the surface morphology of MIL-101 is regular and octahedral. In Figure 1b, after the TEPA is grafted onto the surface of MIL-101, the MIL-101-TEPA retains completely octahedral particles [28]. It can be concluded that the grafting process causes no changes in the morphology of MIL-101. When MIL-101-TEPA is mixed with SA, composite beads are formed. In Figure 1c, MIL-101-TEPA shows aggregation. This phenomenon is attributed to the crosslinking of CA. The EDS in Figure 1d and e shows the distribution of C, N, O, Na, Cl, Ca and Cr throughout the MIL-101-TEPA@CA composite. Cr is characteristic of MIL-101, N is characteristic of TEPA, Na originates in SA, and Ca, Cl originate in CaCl_2_ solution. Therefore, the composite material has been prepared successfully. Figure 1f shows the TEM image of MIL-101-TEPA@CA, MIL-101-TEPA still remains regular and octahedral. The irregular bird nesting part is the crosslinking CA.

#### 3.2.2. FTIR Analysis

**Figure 2:**
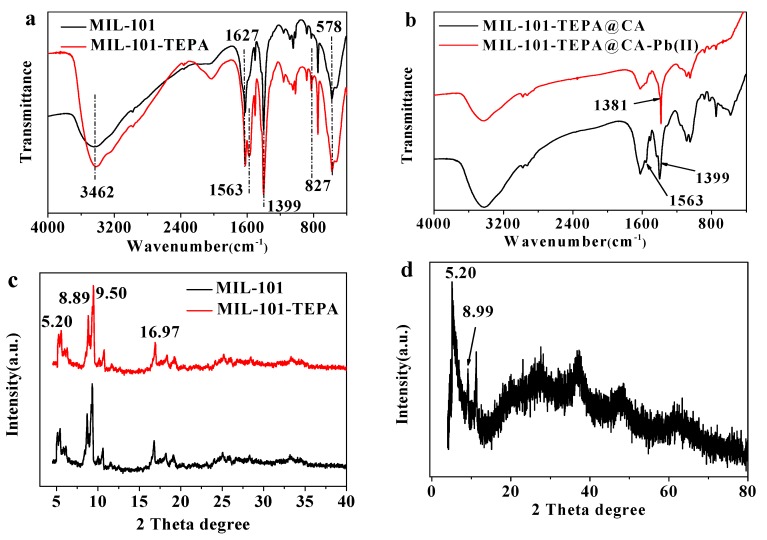
Fourier-transform infrared (FTIR) spectra of (**a**) MIL-101 and MIL-101-TEPA; (**b**) MIL-101-TEPA@CA and MIL-101-TEPA@CA-Pb(II); XRD patterns of (**c**) MIL-101 and MIL-101-TEPA; and (**d**) MIL-101-TEPA@CA.

As shown in Figure 2a, the vibration of O–H leads to the peak at 3415 cm^−1^ appearing in the infrared (IR) spectrum. The two peaks observed at 1627 cm^−1^ and 1399 cm^−1^ can be attributed to symmetric carbonyl stretching vibrations [29]. The vibration of Cr–O causes a deep peak at 578 cm^−1^ [30]. These three characteristic peaks demonstrate the successful synthesis of MIL-101. After TEPA grafting on MIL-101, two new peaks appear in the IR spectrum of MIL-101-TEPA. The peak at 827 cm^-1^ corresponds to the telescopic vibration of –NH_2_, while that at 1563 cm^−1^ is attributed to bending vibrations of N–H in TEPA [31]; this indicates that TEPA is successfully grafted onto MIL-101. According to the IR spectrum of MIL-101-TEPA@CA in Figure 2c, the transmission is decreased; this is attributable to the addition of CA. After Pb(II) loading, the peak caused by the symmetric carbonyl stretching vibrations is moved from 1399 to 1381 cm^−1^. Meanwhile, the peak attributed to the bending vibrations of N–H is weakened. It can be concluded that carboxyl and amino groups participated in the adsorption process.

#### 3.2.3. XRD Analysis

As shown in Figure 2c, XRD patterns of MIL-101-TEPA and MIL-101 show spectral line characteristics typical of crystalline materials. The peaks at 5.20°, 8.89°, 9.50° and 16.97° are characteristic of MIL-101 and we can confirm that the prepared material is MIL-101 [32,33]. Meanwhile, after TEPA is grafted on MIL-101, the XRD pattern is unchanged; this shows that the introduction of TEPA does not destroy the crystallinity. After CA crosslinking with MIL-101-TEPA, the XRD pattern shown in Figure 2d retains the characteristic peaks at 5.20° and 8.89°. The increasing of the signal-to-noise ratio in the XRD pattern conceals any other characteristic peaks.

#### 3.2.4. TGA Analysis

**Figure 3:**
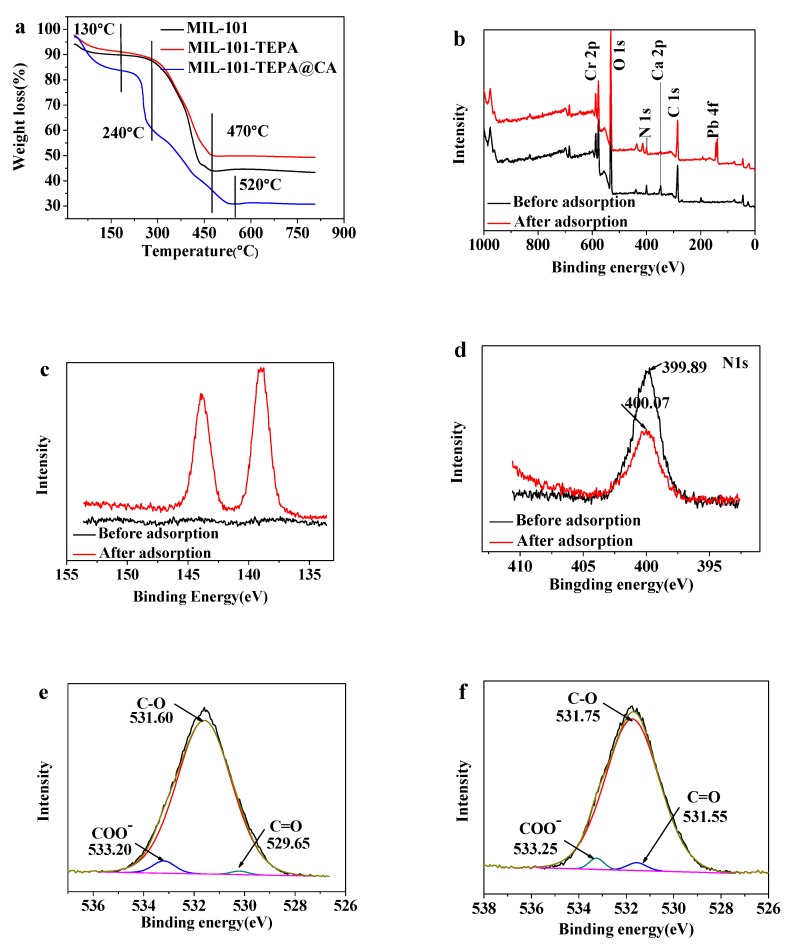
(**a**) Thermal analysis of MIL-101, MIL-101-TEPA, and MIL-101-TEPA@CA; XPS spectra of MIL-101-TEPA@CA before and after loading Pb(II): (**b**) wide-scan; (**c**) Pb 4*f*; and (**d**) N 1*s*, C1*s* spectra (**e**) before and (**f**) after adsorption.

Figure 3a shows the TGA curves of the adsorbents. At temperatures below 130 °C, the weight losses of the three materials of MIL-101, MIL-101-TEPA, and MIL-101-TEPA@CA are 4.95%, 8.35%, and 10.10%, respectively. The weight loss at this stage is mainly due to the evaporation of free water. For temperatures of 240–470 °C, the weight losses of 45.21% and 39.96% for MIL-101 and MIL-101-TEPA, respectively, occur quickly. During this stage, the collapse of the MIL-101 skeleton induces conversion to Cr_2_O_3_, and the weight remains stable. At 240–520 °C, the weight percentage of MIL-101-TEPA@CA is decreased by 49.64%; in this stage, the weight loss process also includes the conversion of CA into CaO. The final residual weight is 30.78%.

#### 3.2.5. XPS Analysis

The XPS spectrum of MIL-101-TEPA@CA in Figure 3b indicates five characteristic elements of Cr, O, C, N and Ca. The first three are the elements comprising MIL-101. Meanwhile, the peak of N 1*s* in Figure 3c indicates that TEPA has been grafted on MIL-101 successfully. The Ca 2*p* peak at 347.1 eV is attributed to the CA precipitate. After the adsorption of Pb(II), an apparent peak of Pb 4*f* appears in the XPS spectrum, demonstrating that MIL-101-TEPA@CA can successfully adsorb Pb(II). As shown in Figure 3d, the binding energy of N 1*s* is 399.89 eV; after loading with Pb(II), it is 400.07 eV. Meanwhile, the absorption intensity is weakened. This attributed to the complexation of Pb(II) with amino groups in TEPA [34]. Figure 3e and f are the C1*s* spectra of MIL-101-TEPA@CA before and after Pb(II) loading. The binding energy peaks at 533.20, 531.60 and 529.65 eV correspond to COO^−^, C–O, and C=O, respectively. After MIL-101-TEPA@CA adsorbs Pb(II), these three binding energies are moved to 533.25, 531.75 and 531.55 eV, respectively. This is caused by the binding of carboxyl and hydroxyl groups to Pb(II).

### 3.3. Optimization of the Adsorption Conditions

#### 3.3.1. Comparison of Adsorption Capacity

**Figure 4:**
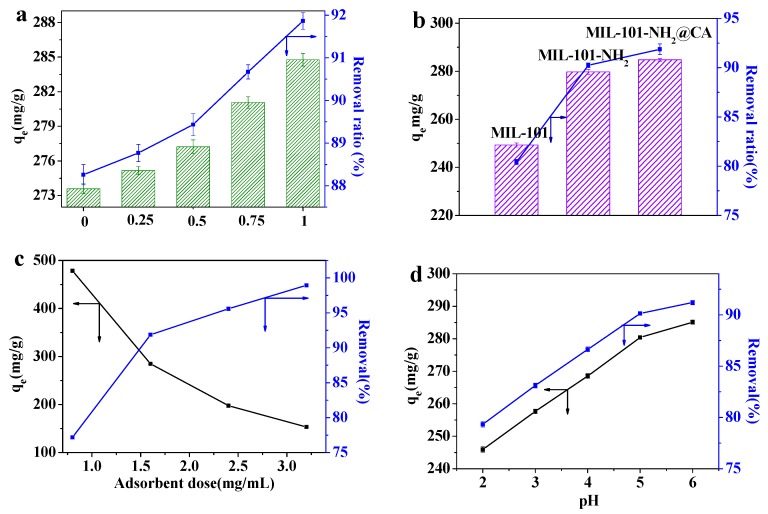
(**a**) The effect of mass ratio for MIL-101-TEPA and CA on the *q_e_*; (**b**) comparison of adsorption capacities of different materials; (**c**) effect of the adsorbent dose on *q_e_*; (**d**) effect of pH on *q_e_*.

The test items were 16.13 mg MIL-101 and MIL-101-TEPA and MIL-101-TEPA@CA with the dry weight equal to 16.13 mg. These three adsorbents were each placed in 10 mL of 500 mg/L Pb(II) solutions. The determined adsorption capacity of each adsorbent is shown in Figure 4b. The *q_e_* of MIL-101 is 249.31 mg/g, while that of MIL-101-TEPA is increased to 279.72 mg/g. CA has a good adsorption capacity for Pb(II) of 273.59 mg/g. However, the mechanical strength of CA is poor, and CA is easily damaged during adsorption. After introducing MIL-101-TEPA, the *q_e_* of MIL-101-TEPA@CA is improved to 284.76 mg/g; simultaneously, the mechanical strength of CA is improved. 

#### 3.3.2. Effect of the Adsorbent Dose

Different weights of MIL-101-TEPA@CA were added to 10 mL 500 mg/L Pb(II) solution to study the relationship between adsorbent dose and *q_e_*. The result is shown in Figure 4c. As the dosage of MIL-101-TEPA@CA is increased, the *q_e_* decreases gradually, because of the increase in Pb(II) near the adsorbent for lower adsorbent concentrations. The utilization of the adsorption sites is high. As the adsorbent concentration increases, the utilization of the adsorption sites decreases, followed by a decrease in adsorption capacity. As the adsorbent concentration increases to 1.613 mg/mL, the Pb(II) removal rate reaches 91.86%. Considering the dosage and removal rate, the optimal adsorbent concentration is determined as 1.613 mg/mL; this concentration is used in the remainder of the experiments.

#### 3.3.3. Effect of the pH of Pb(II) Solution

The surface potential of the adsorbent can be influenced by the pH of the adsorption solution. Adsorbents with stronger electronegativity possess better adsorption capacities for Pb(II). Therefore, pH optimization is necessary for efficient adsorption. When the concentration of Pb(II) solution is 500 mg/L, the pH value is 5.28. Under alkaline conditions, Pb(II) will precipitate as Pb(OH)_2_. HCl and NaOH solutions (0.1 mol/L) were applied to adjust the pH from 2 to 6, to analyze the influence of pH on the adsorption capacity. As shown in Figure 4d, the *q_e_* is increased linearly as the pH increases from 2 to 5. As the pH continues to increase, the adsorption capacity shows a slowing of growth. At lower pH, the carboxyl group is not easily ionized, affecting the combination between the adsorbent and Pb(II). The adsorption capacity is 285.11 mg/g when the pH is 6 and 284.76 mg/g for the solution with unregulated pH. Considering the simplicity of the operation, we determine that it is unnecessary to adjust the pH for adsorption.

#### 3.3.4. Effect of the Initial Concentration of Pb(II)

**Figure 5:**
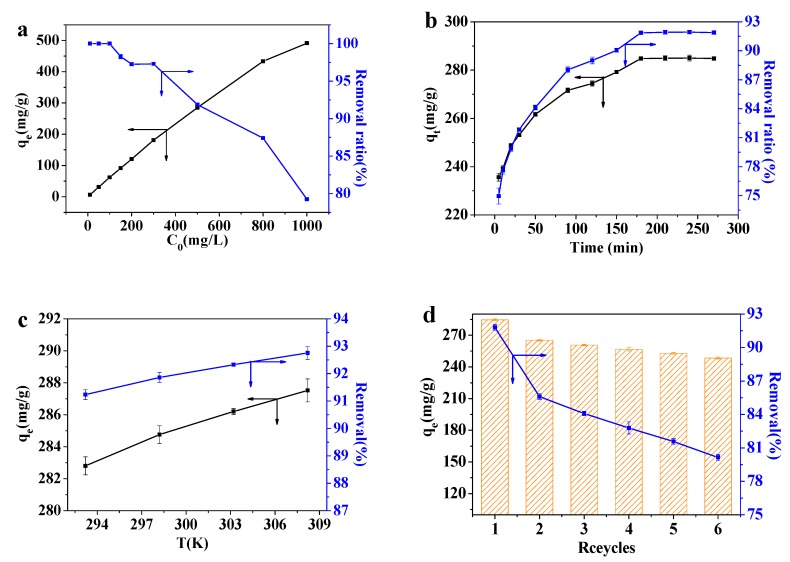
The effect of (**a**) initial Pb(II) concentration; (**b**) adsorption time; (**c**) adsorption temperature, (**d**) adsorption–elution recycling on adsorption capacity.

Pb(II) solutions with concentrations from 10 to 1000 mg/L were used to investigate the relationship between concentration and *C*_0_. The study results are shown in Figure 5a. When the *C*_0_ is below 100 mg/L, the removal ratio reaches 100%. As the *C*_0_ of Pb(II) increases, the removal ratio begins to decrease, but the adsorption capacity continues to increase. With the increase of Pb(II) concentration, the opportunity for binding sites to entrap Pb(II) is increased; thus, the adsorption capacity is increased. At the Pb(II) concentration of 500 mg/L, MIL-101-TEPA @CA can remove more than 90% of Pb(II); meanwhile *q_e_* reaches 284.76 mg/g. Considering the removal ratio, in the following experiments, we choose 500 mg/mL as the experimental Pb(II) concentration.

#### 3.3.5. Effect of the Adsorption Time

The adsorbent (16.13 mg) was dispersed into 10 mL Pb(II) solution for adsorption. The adsorption capacity was measured for each time span. As shown in Figure 5b, when the adsorbing time is in the range 5–90 min, the adsorption capacity is increased quickly. At this time, the concentration of Pb(II) is high and sufficient adsorption sites are available, allowing a high adsorption rate. In the period from 90 to 180 min, the adsorption capacity increases slowly. This is because of the decreases in the quantity of available adsorption sites and the Pb(II) concentration. The adsorption process reaches equilibrium after 180 min, with a total removal rate of 91.86%.

#### 3.3.6. Effect of the Adsorption Temperature

A sample of 16.13 mg MIL-101-TEPA@CA was added to 10 mL of 500 mg/L Pb(II) solution, a series of temperatures from 293 to 308 K were applied to investigate the significance of temperature in the adsorption process. According to Figure 5c, as the adsorption temperature is increased, *q_e_* increases from 282.80 mg/g to 287.52 mg/g, while the removal ratio increases from 91.23% to 92.75%. It can be concluded that a higher temperature favors the adsorption process. The kinematic velocity of Pb(II) is increased at higher temperatures, thus increasing the probability of Pb(II) combination with the adsorbent and thereby increasing the adsorption capacity.

#### 3.3.7. Effect of the Recycling Process

The Pb(II)-loaded MIL-101-TEPA@CA was eluted by 0.1 mol/L HCl. After the desiccation of the eluted MIL-101-TEPA@CA, the *q_e_* of the MIL-101-TEPA@CA was reappraised. The results are shown in Figure 5d. After six adsorption–elution cycles, the adsorption capacity is decreased from 284.56 mg/g to 248.42 mg/g. Although the adsorption capacity is clearly decreased, the adsorption rate still exceeds 80%. This shows the reusability of MIL-101-TEPA@CA.

### 3.4. Adsorption Mechanism

#### 3.4.1. Adsorption Isotherm

We use the Langmuir and Freundlich models to determine the adsorption isotherm. The formula of the Langmuir model is shown in Equation (4); this model is used to explain monolayer adsorption, in which no spatial hindrance occurs [35]. Meanwhile, the Freundlich adsorption isotherm (Equation (5)) is used to describe multilayer and reversible adsorption processes [36]. *R_L_* is a dimensionless separation factor, it can be obtained by the aid of constant *b* in Langmuir model. It is used to determine whether the adsorption occurs preferentially [37].

(4) $$\frac{C_{e}}{q_{e}}=\frac{1}{bq_{m}}+\frac{C_{e}}{q_{m}}$$

(5) $$\mathrm{lg}q_{e}=\mathrm{lg}K_{F}+\frac{1}{n}\mathrm{lg}C_{e}$$

(6) $$\mathrm{lg}q_{e}=\mathrm{lg}K_{F}+\frac{1}{n}\mathrm{lg}C_{e}$$

**Figure 6:**
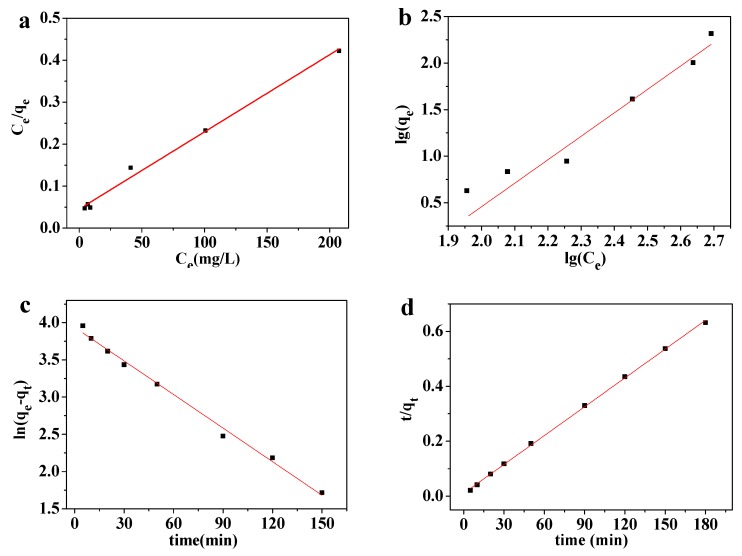
Fitted curves obtained by the (**a**) Langmuir and (**b**) Freundlich isotherm models, (**c**) the pseudo-second-order and (**d**) the pseudo-first-order kinetics models for the adsorption of Pb(II) by MIL-101-TEPA@CA.

Here, the maximum adsorption capacity *q_m_* (mg/g) can be obtained by the Langmuir model, *b* is a constant of Langmuir model; *n* is a constant of the Freundlich adsorption isotherm relating to the adsorption intensity, while *K_F_* is a constant representing the adsorption capacity. Figure 6a,b show the fitting results of the Langmuir and Freundlich models, and the related parameters are shown in Table 1. The correlation coefficient (*R*^2^) of the Langmuir model is 0.9910, while that of the Freundlich model is 0.9473; the adsorption process is more suitably fitted by the Langmuir model. This indicates that the adsorption sites are uniformly distributed on the surface of MIL-101-TEPA@CA. The *q_m_* fitted by the Langmuir model is 543.48 mg/g. The values of *R_L_*, as shown in Table 2, are all less than 1, indicating that the process of MIL-101-TEPA@CA for Pb(II) is energetically favorable. The *q_m_* and adsorption mechanism of MIL-101-TEPA@CA for Pb(II) are compared with those of previously reported adsorbents, as summarized in Table 3. It can be concluded that MIL-101-TEPA@CA is the most promising adsorbent [38].

#### 3.4.2. Adsorption Kinetics Study

The pseudo-first-order kinetics model (Equation (7)) is applied to describe the adsorption process in which the adsorbate attachment to the surface of the adsorbent is controlled by diffusion [45]. The pseudo-second-order kinetics model (Equation (8)) is applied to describe adsorption processes where the adsorbate attachment to the adsorbent surface is controlled by chemisorptions [46].

(7) $$\ln(q_{e}-q_{t})=\ln q_{e}-k_{1}t$$

(8) $$\frac{t}{q_{t}}=\frac{1}{k_{2}q_{e}^{2}}+\frac{t}{q_{e}}$$

Here, *k*_1_ (1/min) and *k*_2_ (g/mg∙min) are the pseudo-first-order kinetics model and the pseudo-second-order kinetics model rates, respectively. The fitting curves and the related parameters are shown in Figure 6c–d, and Table 4, respectively. The correlation coefficient (*R*^2^) of the pseudo-first-order kinetics model is 0.9930; that of the pseudo-second-order kinetics model is 0.9994. The adsorption process has a high degree of conformance to the pseudo-second-order kinetics model, and *q_e_* fitted by the pseudo-first-order kinetics model is 285.71 mg/g, near the adsorption capacity obtained experimentally. Therefore, the adsorption process likely occurs by chemical adsorption.

#### 3.4.3. Adsorption Thermodynamics Study

The relevant thermodynamic parameters are obtained by Equation (9). The adsorption mechanism of MIL-101-TEPA@CA for Pb(II) was further studied using the thermodynamic parameters.

$$\Delta G=-RT\ln\frac{q_{e}}{C_{e}}=-RT(-\frac{\Delta H}{RT}+\frac{\Delta S}{R})$$

Here, *ΔG* (kJ/mol) is the Gibbs free energy, *ΔH* (kJ/mol) is the enthalpy, *ΔS* (kJ/mol) is the entropy, *T* (K) is the thermodynamic temperature, and *R* is the gas constant. The related parameters are shown in Table 5. After substituting the known parameters *q_e_* and *C_e_* at different temperatures into Equation (5), *ΔG* is negative, so the adsorption process is an endothermic reaction. In addition, *ΔH* is positive, further confirming the above conclusion. Therefore, a relatively high temperature benefits the adsorption process. *ΔS* is also negative, so the adsorption of Pb(II) by MIL-101-TEPA@CA is an entropy-driven process.

## 4. Conclusions

In this study, MIL-101-TEPA@CA shows a good ability to remove Pb(II) in liquids. The introduction of TEPA greatly enhances the removal ratio of MIL-101-TEPA for Pb(II). The fabricated beads of MIL-101-TEPA@CA can be easily recovered from treated liquids by simple filtration. The effects of the adsorbent dose, adsorption time, initial concentration of Pb(II), pH of the Pb(II) solution, and adsorption temperature on the adsorption process have been studied. The study of MIL-101-TEPA@CA for Pb(II) proves that the adsorption process occurs following the pseudo-second-order kinetics model and the Langmuir adsorption isotherm. The time of adsorption equilibrium is 180 min; after six cycles of reuse, the removal ratio remains above 82%. In summary, MIL-101-TEPA@CA as an adsorbent shows a high adsorption capacity and good recyclability.

## Figures and Tables

**Table 1:** Parameters of langmuir and freundlich models for Pb(II) removing by MIL-101-TEPA@CA.

*T* (K)	Langmuir Isotherm			Freundlich Isotherm		
	*q_m_*(mg/g)	*b* (L/mg)	*R* ^2^	*K_F_*	*n*	*R* ^2^
298.2	543.48	0.040	0.9910	1.27×10^−4^	0.38	0.9473

**Table 2:** *R_L_* for Pb(II) Removal by MIL-101-TEPA@CA Based on the Langmuir Model.

***C*_0_ (mg/L)**	10	50	100	200	300	500	800	1000
***R_L_***	0.67	0.29	0.17	0.091	0.063	0.038	0.024	0.020

**Table 3:** Comparison of the maximum adsorption capacity for Pb(II) with reported adsorbents.

Adsorbents	*q_max_*_(mg/g)_	Adsorption Mechanism	References
NH_2_-MIL-101(Cr)	81.09	electrostatic interaction	[39]
POP-NH_2_	523.6	coordinated complexation	[40]
NFC/PEI	357.14	chemical adsorption	[41]
Cellulose 4	192.3	coordinated complexation, electrostatic interaction	[42]
Al(OH)_3_/(PAA-CO-PAM)	106.2	chemical adsorption	[43]
Cs-PMA/HNT	357.38	coordinated complexation, electrostatic interaction	[44]
MIL-101-TEPA@CA	543.48	coordinated complexation, electrostatic interaction	this work

**Table 4:** Kinetic models parameters for the Pb(II) removal by MIL-101-TEPA@CA.

*q_e, exp_*	Pseudo-First-Order Model			Pseudo-Second-Order Model		
	*q_e, cal_*	*k*_1_	*R* ^2^	*q_e, cal_*	*k*_2_	*R* ^2^
284.76	10.70	0.02390	0.9930	285.71	0.0011	0.9994

**Table 5:** Thermodynamics parameters of MIL-101-TEPA@CA for removing Pb(II).

ΔG (kJ/mol) *T* (K)				ΔH (kJ/mol)	ΔS (J/mol∙K)
293.2	298.2	303.2	308.2		
−4.54	−4.82	−5.07	−5.31	10.30	−0.051
